# The effects of narrative versus non-narrative information in school health education about alcohol drinking for low educated adolescents

**DOI:** 10.1186/s12889-015-2425-7

**Published:** 2015-10-23

**Authors:** Simon Zebregs, Bas van den Putte, Anneke de Graaf, Jeroen Lammers, Peter Neijens

**Affiliations:** The Amsterdam School of Communication Research ASCoR, Department of Communication Science, University of Amsterdam, Nieuwe Achtergracht 166, 1018 WV Amsterdam, The Netherlands; Trimbos Institute, Netherlands Institute for Mental Health and Addiction, Postbus 725, 3500 AS Utrecht, The Netherlands; Centre for Language Studies, Faculty of Arts, Radboud University Nijmegen, Postbus 9103, 6500 HD Nijmegen, The Netherlands

**Keywords:** Adolescence, Alcohol, Drinking behaviour, Health education, Students, Narrative/non-narrative information

## Abstract

**Background:**

Traditionally most health education materials are written in an expository non-narrative format. Scholars have argued that the effectiveness of materials may increase when these texts are replaced by narrative texts, and that the non-narrative texts should be replaced by narrative texts. However, no previous studies have tested these claims in the context of school health education for low educated adolescents. This study aims to do so for an existing preventive health education intervention about alcohol for low educated adolescents. Based on the empirical findings of previous studies, it is expected that the claims about narratives being more effective than non-narrative texts are not true for effects on knowledge. Instead non-narrative texts are expected to have a stronger impact on this outcome variable. For attitude towards alcohol and intention to drink alcohol the claims are expected to be true, because participants are expected to be less aware of the persuasive intent of the narrative texts, which would make them less resistant. As a result, narrative texts are expected to have a stronger effect on attitude and intention.

**Methods:**

This study compares the effects on knowledge, attitude towards alcohol, and intention to drink alcohol of both information formats in a two-condition (non-narrative vs. narrative information) experiment with repeated measures (pre-measurement, immediate post-measurement, and delayed post-measurement). The experiment was conducted amongst 296 students of the two lowest levels of the Dutch secondary education system.

**Results:**

The results showed immediate effects on knowledge and attitude towards alcohol, which did not differ between conditions and school levels. These effects did not persist over time. There were no effects on intention to drink alcohol.

**Conclusion:**

It is concluded non-narrative and narrative information are equally effective in the context of school health education, suggesting the claims that scholars have made about the superior effects of narrative texts are not true. Given the fact that narrative texts are more expensive to develop, policy makers may not be advised to prefer these types of texts over the traditionally used non-narrative texts.

## Background

Education level is associated with various health disparities, such as higher mortality risk [1], poorer oral health [2], higher risk of activity limitation due to chronic diseases [3], and higher chances of alcohol abuse, tobacco use, and drugs use [4]. Health education materials are often applied to target these health disparities. However, research on strategies to make such materials most effective is often only conducted amongst higher educated target groups. This is a problem, because education level is associated with cognitive capacities in a way that lower educated people generally have fewer cognitive capacities (e.g, [5, 6]). This makes them more likely to experience difficulties while processing information from health education materials [7]. Hence, for lower educated target groups it is particularly important that materials are designed in such a way that these facilitate information processing in a most optimal manner.

Most often texts in health education are written in a expository non-narrative format, but suggestion have been made that replacing these texts with narratives would make materials more effective [8, 9]. Non-narrative information is presented in an abstract, general manner, using logical reasoning and factual information [10]. Narrative information, on the other hand, contains cohesive stories describing a setting and episode from the perspective of one or more protagonists, often providing information about goals, plans, actions, and outcomes [11]. Using narrative information is suggested to make texts more enjoyable [12]. As a result, the use of narrative information could help to make the educational material less of a “burden” and therefore increase students’ motivation to read it [13]. This may be particularly relevant for low educated students, because information processing, and consequently learning, is more cognitively demanding for them than for high educated students [7]. However, an important question is how the effects of the narrative information format in health education materials relate to the effects of the non-narrative information format that is traditionally used. Scholars have suggested that narrative information may be superior to non-narrative information in a way that it merits the additional costs that are involved with the development of materials containing narrative information [9]. Moreover, narrative information formats have been successfully tested in education-entertainment formats, often distributed via entertainment media (e.g, [14, 15]). However, the effects of narrative and non-narrative information have not been compared in the context of health education for low educated people before. In addition, although beliefs in the literature about persuasive effects appear to be consistently in favour of narratives, for learning outcomes there are also suggestions in favour of using non-narrative texts [16, 17]. Hence, it is unknown whether narrative or non-narrative information in health education materials has the strongest impact on low educated people and whether this differs across outcome variables.

In this study we will focus on the role of information formats in health education materials about alcohol from the Dutch “healthy education and drug” program. The program focusses on prevention, which means that it targets students that do not yet drink alcohol. Dutch schools are not obligated to educate students about alcohol. Nevertheless, the “healthy school and drugs” program is estimated to be implemented at 70 % of the Dutch secondary schools. The program consist of an information component that focuses on the consequences of drinking alcohol and a skills training component that focuses on the skills required to resist negative social influences. It is developed based on the I-Change model [18]. The model assumes that people first need to be aware of the negative consequences of a behaviour, and then need to develop a motivation (intention) not to engage in the behaviour. Intention is amongst other determinants based on people’s attitude towards the behaviour, which is in turn based on people’s perception of the positive and negative consequences of the behaviour. These determinants are the focus of the information part of the intervention. Eventually, people’s motivation needs to turn to action, for which people require specific skills. In case of the current intervention this means that adolescents need to refrain from drinking alcohol and for this purpose they require the skills to resist negative social influences. This is the focus of the skills training component of the program.

The existing information materials from this program have been written in an expository non-narrative format with the aim to let adolescents consider the consequences of drinking alcohol and adjust their attitudes and intention to drink alcohol accordingly. These materials are applied in the first year of the Dutch secondary school system (seventh grade) and are found to be effective for increasing knowledge about the consequences of drinking alcohol amongst the general population of Dutch secondary education students [19]. In this study, we will focus on students from the lower education levels and whether effectiveness of existing materials increases when the non-narrative texts are replaced by narratives. We specifically focus on the information component of the program, because the skills training component only contains exercises and does not include any texts that could be written in a narrative format. Effectiveness is examined through the most important outcome measures of these materials, being knowledge about the negative consequences of drinking alcohol, attitude towards alcohol, and intention to drink alcohol. The effects in this study will be examined immediately after exposure and approximately four weeks later. Through this study we make an important contribution to the existing knowledge about the usage of different information types in health education.

### Learning effects

While research has shown that people can learn from narrative information [20], the current literature offers contradicting views on whether narrative information can be expected to have a stronger learning effect than non-narrative information [13]. On the one hand, narrative information is suggested to be effective by modelling behaviour and allowing for vicarious learning, so people can experience what the consequences of behaviour are without performing it themselves [21]. As such, narrative information is more vivid than non-narrative information in which the consequences would be described in a more abstract manner [22]. Vividness is considered to establish more divers associations in memory [23]. Information that is stored in memory with more associations is more likely to be activated and retrieved when needed [24, 25]. Therefore, narrative information could have a stronger learning effect than non-narrative information. On the other hand, it is also suggested that in an educational context narrative information could have a seductive details effect that would make it less effective than non-narrative information [13, 16]. According to the seductive details hypothesis, narratives contain interesting but irrelevant details. Such details may distract students’ attention from the information that is relevant. As a result, they may remember less of the relevant details [26, 27].

In this study we will specifically examine the effects of written materials. The impact of written materials is likely to differ from the impact of film, because written materials require more visualizing and imagination (e.g, [28]). Therefore, we will focus on the results of previous studies that applied written texts. To our knowledge, only two studies compared narrative with non-narrative information in written school education materials amongst adolescents. A study conducted amongst Dutch pre-vocational students found evidence that supported the seductive details hypothesis [16]. These findings are similar to these of a study conducted amongst American high school students that showed a stronger learning effect when a non-narrative text was read instead of a narrative text [17]. Studies conducted amongst college students showed more mixed results [29, 30]. One study found that participants recalled more information from the texts after reading a non-narrative text, which is in line with the findings of research conducted amongst adolescents [30]. However, another study found narrative texts to have a stronger effect on knowledge amongst participants with low prior knowledge, and non-narrative texts to have a stronger effect on knowledge amongst high prior knowledge participants. This study did not find any main effects [29]. Despite the mixed results of studies amongst college students, the overall findings of previous research seem to suggest that non-narrative texts have a stronger learning effect. Therefore, although the previous studies did not examine health information, by lack of other previous evidence we hypothesize that non-narrative information will have a stronger learning effect than narrative information for school health education material as well.

H1: School health education materials containing non-narrative information have a stronger effect on knowledge than school health education materials containing narrative information.

### Persuasive effects

Although we expect non-narrative information to have a stronger learning effect than narrative information, this does not imply that similar effects can be expected on attitude and behavioural intention. Persuasion is suggested to involve a special form of learning. This means that the information that people learn needs to be integrated with a person’s current beliefs. If the newly learned information contradicts these beliefs, this information may be disregarded [31]. Hence, it is more difficult to persuade people than to educate them and materials that have a stronger effect on knowledge do not necessarily have a stronger persuasive effect.

In the case of the comparison between narrative and non-narrative information it is likely that non-narrative information will have a stronger learning effect, while narrative information has a stronger persuasive effect. It is suggested that people generally consume narrative information with the goal to get entertained [32]. While having this goal, people are presumed to get engaged with the story and to identify themselves with the characters in the narrative [33]. As a consequence, they are assumed to pay less attention to the persuasive intentions behind the message and engage in less critical processing. This makes them more likely to accept the knowledge they learned instead of disregarding it based on contradictions with their existing beliefs [32].

Based on the theoretical assumptions above, it could be expected that school health education materials about substance use are more persuasive when they contain narrative instead of non-narrative information. To our knowledge, there are no previous studies that have compared the persuasiveness of narratives with non-narratives in school health education materials, although two studies did make this comparison with written materials in the context of health promotion messages. One study about skin cancer prevention was conducted amongst college students [10]. This study contained conditions with narrative information or non-narrative information as well as a control condition without any information. The results showed that students in the narrative condition engaged in significantly more self-examination and information searching, and talked significantly more about skin cancer to their family than students in the control condition. Students in the non-narrative condition only engaged in significantly more information searching than students in the control condition. There were, however, no significant differences between the narrative and the non-narrative condition. In another study Janssen and colleagues [34] found that sunbed users felt significantly more vulnerable after receiving a narrative message than after receiving a non-narrative message. In line with the theoretical assumptions, these two studies showed some, although weak, evidence that narrative information is more effective than non-narrative information.

In our study we will focus on students’ attitude towards drinking and intention to drink alcohol, as these are perceived to be the important determinants of their future behaviour [35]. Although previous studies on narrative versus non-narrative health information have not examined effects on health behaviour attitude, based on theory and the scarce evidence, we expect narrative information to have a stronger effect on this variable.

H2: School health education materials containing narrative information have a stronger effect on attitude towards alcohol than school health education materials containing non-narrative information.

Finally, in line with the results of previous research, we expect narrative information to have a stronger impact on students’ intention to drink alcohol than non-narrative information.

H3: School health education materials containing narrative information have a stronger effect on intention to drink alcohol than school health education materials containing non-narrative information.

## Methods

At Dutch secondary schools we conducted a three-wave experiment with two conditions (non-narrative information vs. narrative information). The experiment included a pre-measurement (T1), an immediate post-measurement (T2), and a delayed post-measurement (T3). The interval between waves was approximately four weeks.

### Materials

To manipulate the information format the stimulus materials existed of two booklets, which were based on existing health education materials from the Dutch Trimbos Institute’s “Healthy School and Drugs” program. One booklet contained information about alcohol in a non-narrative form (treatment-as-usual), while the other contained the exact same information in a narrative form. Each booklet contained five pages of texts addressing fifteen negative consequences of alcohol consumption and five pages of exercises. Narrative information naturally contains information about the setting and perspective of the protagonists, and provides information about actions, and outcomes, whereas non-narrative information does not [10, 11]. Consequently, the average number of words per page differed between the narrative (*M* = 149.20; *SD* = 10.92) and non-narrative information (*M* = 64.40; *SD* = 13.83) condition. Examples of texts from both conditions are provided below.

Example non-narrative text: *When you drink alcohol, it makes you feel different. You can become happier. When drinking alcohol you also may dare more. Then you can start to do risky things. However, due to the alcohol, your control over your physical movements will decrease. This makes you more likely to fall down.*

Example narrative text: *Patrick, Lisa, and Angelo are sitting on a bench in the park. Patrick brought some cans of beer and asks: “Who wants a beer?” Lisa responds: “I do!” She is curious. Angelo does not respond at all. He doesn’t want to drink alcohol. Lisa opens a can and takes a few sips. Patrick immediately finishes a whole can. Then he takes another one, and another one. He starts to feel different. The alcohol makes him feel happy. Patrick also dares to do more risky things. He climbs on one of the other benches and walks on the edge of the back. Due to the alcohol Patrick has less control over his movements. He loses his balance and falls on the ground. He stands up and says to the others: “Let’s go home.”*

In both booklets there were fully identical exercises following each text page. These exercises are part of the regular intervention that we used for the treatment-as-usual condition. The exercises consist of an exercise that lists eight drinks for which students need to indicate whether these contain alcohol, five “yes or no” questions about the effects of alcohol, two multiple choice questions, and an exercise in which students get six pieces of information from which they need to pair the ones that are related. The intervention includes these exercises, because education literature prescribes the use of exercises in educational materials to provide students with opportunity to process information from the texts. Without this break in the provision of information, students may experience an overload that hinders the storage information [36].

### Sample size

We estimated a required sample size based on a small between conditions effect of Cohen’s f = .10. Meta-analyses have shown that studies on message factors generally find small effect [37]. We conducted a power calculation for a repeated measures analysis of variance with within-between subject interaction with three waves, two conditions, and two school levels, assuming a .5 correlation between repeated measures. The calculation revealed that given an alpha of .05 and a required power of .80, we would require a minimum total sample of 232 participants.

### Participants

First year students of both special education schools and pre-vocational schools participated in this study (age 11–14). Special education schools form a level below the lowest mainstream level of the Dutch secondary education system. This school level is intended for students with severe learning difficulties who are only allowed to enrol if they meet special requirements, of which an IQ (Intelligence Quotient) between 60 and 75 is the most important one. Because students at special education schools require much individual attention, groups usually contain ten students at most. The aim at these schools is to prepare students for the job market. Pre-vocational schools form the lowest mainstream level of the Dutch secondary education system. Within the pre-vocational schools we sampled particularly classes that included students within a special support program. Students in this program receive additional support, because they have learning difficulties that disable them to perform at a sufficient level without this additional support. These students can enrol in the support program after receiving advice from their school. Students usually continue to enrol in vocational education after finishing pre-vocational school.

Seven special education schools were invited, which were existing contacts of our research group. These schools all agreed to participate. No existing contacts were available amongst pre-vocational school. Hence, 122 schools of this type were invited from across the country. Five of these schools agreed to participate. In total 171 special education school students, divided over 15 groups from seven schools, and 184 pre-vocational school students, divided over 12 groups from five schools, entered the randomization process. The average group size was slightly below 14 (*M* = 13.77; *SD* = 5.10).

A sealed envelope procedure was applied by the first author to allocated groups to one of the two conditions. Randomization of groups was preferred over randomization of individual participants, because data was collected in a classroom setting. Within this setting participants could become aware that they received different booklets, which made it undesirable to have booklets from the two conditions in one group. During the randomization procedure a stack of 28 identical looking envelopes was prepared, which contained a note with the name of one of the conditions. These envelopes were shuffled thoroughly and one envelope was assigned to each of the included groups. Because there were 27 groups to assign, one envelope remained unassigned.

**Fig. 1:**
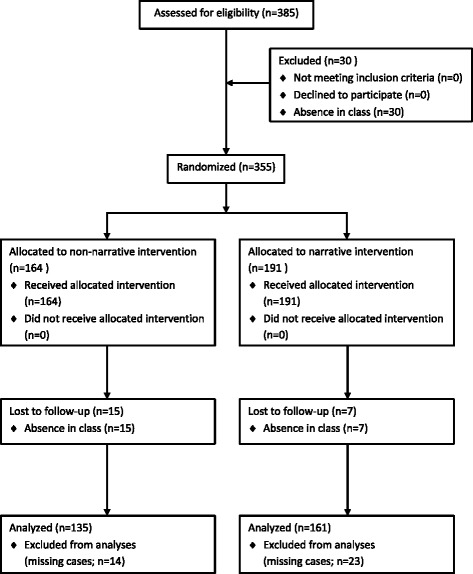
Flow diagram

Eventually, 158 special education school students and 159 pre-vocational school students completed all three waves. The students who dropped out at special education schools were equally divided over the conditions (*n* = 6 per condition). At pre-vocational school level more students dropped out in the narrative information condition (*n* = 17) than in the non-narrative condition (*n* = 8). All dropouts were caused by the absence of students in class due to illness or other obligations. The higher number of dropouts in the narrative information conditions at pre-vocational schools was due to the same reasons. At T1 the dropout students had significantly more experience with drinking alcohol (44.4 %) than the students who completed all three waves (30.8 %; *χ*^2^ = 15.83; *p* = .045). Before the analyses 13 special education school students and eight pre-vocational school students were removed from the sample because of missing values. Our final sample contained 145 special education school students and 151 pre-vocational school students (see Fig. 1 for the flow diagram).

The sample contained a nearly equal number of boys and girls. On average the students were twelve and a half years old. The largest share of participants was born in the Netherlands, and spoke mainly Dutch at home. About half of the participants indicated to have no religion. The largest groups of religious participants in our sample were Catholics and Muslims. Chi-square tests indicated that these numbers did not differ between conditions or school levels (*p*’s > .05; see Table 1).

**Table 1:** Overview of demographics: gender, age, gender, country of birth, primary language at home, and religion per group

	Non-narrative (*n* = 135)	Narrative (*n* = 161)
Gender		
Boys	54.1 %	48.4 %
Girls	45.9 %	51.6 %
Age		
11	2.2 %	2.5 %
12	48.1 %	52.3 %
13	48.1 %	44.1 %
14	1.5 %	1.2 %
Country of birth		
Netherlands	95.5 %	92.5 %
Turkey	1.5 %	0.0 %
Other	3.0 %	7.5 %
Primary language at home		
Dutch	89.6 %	83.1 %
Turkish	3.7 %	1.9 %
Arab	3.0 %	2.5 %
Berber	0.7 %	2.5 %
Papiamento	3.0 %	9.4 %
Other	0.0 %	0.6 %
Religion		
No religion	49.6 %	51.9 %
Catholic	27.5 %	28.7 %
Protestant	7.6 %	1.9 %
Muslim	9.9 %	13.8 %
Hindu	0.8 %	1.9 %
Buddhist	0.8 %	1.3 %
Jewish	1.5 %	0.0 %
Other	2.3 %	9.6 %

### Ethics statement

The ethical committee of the Faculty of Social Sciences at the University of Amsterdam approved this study. The study was classified in the low risk category. Previous research on the “healthy school and education” program did not reveal any unintended effects on knowledge about alcohol, attitude towards alcohol, and drinking behaviour [19]. In accordance with the procedures of the ethical committee, we first informed schools about the details of the study and did not continue until they formally agreed to participate in writing. Before the study started, parents were informed about the participation of their child through an information letter. Attached to the information letter was a form that parents could sign and return if they did not agree with their child’s participation. This method of consent is in accordance with the rules specified in the ethical committee’s guidelines and explicitly agreed on following the ethical committee’s review of the study protocol (registration number: 2013-CW-11). The rejection rate was below five percent.

### Procedure

All data for this study were collected in a classroom setting. At the first wave, the students received instructions as a group, after which they individually completed the questionnaire. The questionnaire contained items about the dependent and control variables and took about 25 min to complete.

The second wave took place about four weeks after the first wave. At this wave classes were randomly assigned to one of the conditions. There were slightly more students in the narrative condition (*n* = 161) than the non-narrative condition (*n* = 135). At the start of the session of the second wave, students received instructions as a group. Hereafter, they individually completed the booklet of the condition they were assigned to, which took about 15 min. After finishing the booklet, students handed it in and received the questionnaire. It took the students about 20 min to complete the questionnaire, which contained items about the dependent variables.

Finally, the third wave took place about four weeks after the second wave. After receiving instructions as a group, the students completed a questionnaire. This questionnaire took about 20 min for the students to complete.

During all waves the data collection was administered by the first author of this article, in collaboration with the teachers of the various groups. The teachers were present at all time to maintain a setting that was as normal as possible. At the time of the study, the first author (male) had a master’s degree and one year of experience with instructing groups in this type of research settings. He provided instructions to one group at the time and stayed present in the classroom while students read the booklet and responded to the questionnaires. Through this approach it was possible to have the intervention delivered to each group in the way it was intended.

### Measures

The participants responded to items about several determinants of drinking behaviour at each wave. We adapted all measures to the abilities of the low educated adolescents in our samples. Below we describe the measures that we included in our analyses.

#### Knowledge

To measure knowledge we used an open-ended question, which asked the participants to write down as many negative consequences of drinking alcohol that they knew. This measure was validated in previous research [38] and has been applied in a previous study amongst students form grade 7 till 9 [39]. Two coders coded the validity of responses, meaning they coded whether each response was indeed a negative consequence and unique (i.e., only listed once). The inter-coder reliability was examined by computing the correlation between coders at each wave (T1: *r* = .98; *p* < .001. T2: *r* = .98; *p* < .001. T3: *r* = .99; *p* < .001.). In case of differences between coders the responses were examined and discussed until agreement was reached. The number of negative consequences was used as a measure of knowledge (T1: *M* = 3.08; *SD* = 1.50. T2: *M* = 3.64; *SD* = 1.60. T3: *M* = 2.95; *SD* = 1.46).

#### Attitude towards alcohol

At each wave attitude towards alcohol was measured through five semantic differential items, based on Ajzen [40]. The sentence “I find alcohol drinking…” was followed by the items: *negative-positive, unenjoyable-enjoyable, unwise-wise, unpleasant-pleasant,* and *bad-good.* All items were measured on a four-point scale ranging from 1 to 4. We averaged all items per wave to create an indicator of attitude towards alcohol (T1: α = .92; *M* = 1.77; *SD* = .71. T2: α = .92; *M* = 1.65; *SD* = .67. T3: α = .93; *M* = 1.71; *SD* = .70).

#### Intention to drink alcohol

We measured intention to drink alcohol at each wave through the statements “I plan to drink alcohol”, “I plan to drink alcohol in the upcoming month”, and “I plan to drink alcohol in the upcoming year” [40]. Participants responded through a four-point scale ranging from 1 (*no, certainly not*) to 4 (*yes, certainly*). We averaged all statements per wave to create an indicator of intention to drink alcohol (T1: α = .76; *M* = 1.67; *SD* = .68. T2: α = .81; *M* = 1.65; *SD* = .70. T3: α = .82; *M* = 1.71; *SD* = .74).

#### Control variables

To measure students’ past behaviour we included two questions at T1 to control for possible differences in behaviour between conditions and school levels. First, we measured how often students consumed alcohol through a closed-ended question with the response categories *never, 1–3 days per year, 4-days per year, 1 day per month, 2–3 days per month, 1 day per week, and more than 1 day per week*. Second, we asked the students on how many days they had consumed alcohol during the past month.

#### Additional variables

Additional analyses were conducted to compare the perception of persuasive intent and levels of counterarguing between conditions and school levels. Both measures were based on one item and had a four-point scale (1 *No, not at all* – 4 *yes, very much*). The item for perceived persuasive intent was formulated as “*The writer of the text wants to prevent me from drinking alcohol*” (*M* = 3.11; *SD* = .97). Counterarguing was measured with the item “*While reading the text I had positive thoughts about alcohol*” (*M* = 1.88; *SD* = .89).

### Analyses

In this study we randomly assigned groups to conditions. Hence, the data was nested within these groups. We examined whether the variance within these groups was of such proportions that it should be taken into account by calculating intraclass correlation values for all dependent variables. If the intraclass correlation value would exceed .05, then multilevel analysis would be required [41]. This did not apply to any of the dependent variables. Therefore, we examined all our hypotheses through repeated measures analyses of variance in SPSS 20. In all analyses we included information type (non-narratives vs. narratives) and school level (special education vs. pre-vocational schools) as between-subjects factors. Contrasts were computed using a Bonferroni correction for multiple comparisons. In addition, we used Chi-square tests to examine any differences in past behaviour between school levels and conditions, and one-way ANOVA tests to conduct additional analyses for counterarguing and perceived persuasive intent.

## Results

At the baseline measurement, on average the students knew about three negative consequences of consuming alcohol, had a slightly negative attitude towards alcohol, and a low intention to consume alcohol (see Table 2). In addition, most of the participants indicated that they never consumed alcohol (*n* = 197; 66.6 %). The largest share of the participants who consumed alcohol indicated to do this at one to ten days per year (*n* = 64; 21.6 %), while about one out of ten students consumed alcohol once a month or more (*n* = 26; 8.8 %). Some of the students who participated in this study indicated to have consumed alcohol in the past month (*n* = 51; 16.0 %). In most cases this was on one day (*n* = 30; 9.5 %). Chi-square tests indicated that there were no differences in previous behaviour between conditions or school levels (*p*’s > .05).

**Table 2:** Estimated means and standard deviations of knowledge, attitude towards alcohol and intention to drink alcohol per wave, and effect sizes for differences between conditions in changes between waves

	*M* (*SD*)			ES for differences between conditions Cohen’s d		
	T1	T2	T3	T1-T2	T1-T3	T2-T3
Knowledge				-.14	.07	.20
Non-narrative	3.13 (1.41)^a^	3.76 (1.65)	2.94 (1.52)^a^			
Narrative	3.14 (1.56)^b^	3.57 (1.51)	3.05 (1.40)^b^			
Overall	3.14 (1.49)^c^	3.66 (1.57)	3.00 (1.46)^c^			
Attitude towards alcohol				-.07	-.04	.03
Non-narrative	1.76 (.69)^d^	1.67 (.68)^d^	1.72 (.69)^d^			
Narrative	1.81 (.74)^e^	1.67 (.67)^f^	1.74 (.71)^e,f^			
Overall	1.79 (.72)^g^	1.67 (.67)	1.74 (.70)^g^			
Intention to drink alcohol				-.07	-.01	-.08
Non-narrative	1.68 (.66)^h^	1.68 (.69)^h^	1.72 (.70)^h^			
Narrative	1.71 (.71)^i^	1.66 (.71)^i^	1.74 (.77)^i^			
Overall	1.69 (.69)^j^	1.67 (.70)^j^	1.73 (.74)^j^			

*Note*. Superscript indicates group means that do not differ significantly (*p* > .05) between waves according to the Bonferoni corrected post-hoc test

### Main analyses

Our first hypothesis predicted that school health education materials containing non-narrative information would have a stronger effect on knowledge than school health education materials containing narrative information. The results showed that averaged over both conditions knowledge changed significantly over time (*F* (2,584) = 24.65, *p* < .001, η^2^ = .078). Contrasts revealed that students had significantly more knowledge at T2 than at T1 and at T3, but there was no difference between T1 and T3 (see Table 2). The interaction test between time and information type revealed that this learning effect did not differ between conditions (*F*(2,584) = 1.13, *p* = .323, η^2^ = .004). School level, which we controlled for in our analyses, had no influence on any of these effects (*p*’s > .05). Based on these findings we conclude that the health education materials had an equally strong immediate effect on knowledge in both conditions that did not persist until T3. Hence, our first hypothesis was rejected.

Hypothesis 2 predicted that school health education materials containing narrative information would have a stronger effect on attitude towards alcohol than school health education materials containing non-narrative information. For attitude, there was a significant main effect of time (*F* (2,584) = 8.52, *p* < .001, η^2^ = .028). Contrasts showed that students had a significantly more negative attitude towards alcohol at T2 than at T1 and at T3, while there was no difference between T1 and T3 (see Table 2). There was no significant interaction between time and information type (*F* (2,584) = .32, *p* = .729, η^2^ = .001), which indicates that there was no difference between conditions. None of the effects were influenced by school level (*p*’s > .05). We conclude that similar to knowledge there was an equally strong immediate effect on attitude in both conditions, which did not persist over time. Therefore, our second hypothesis was rejected.

Finally, our third hypothesis predicted that school health education materials containing narrative information would have a stronger effect on intention to drink alcohol than school health education materials containing non-narrative information. Unlike students’ knowledge and attitude, intention to drink alcohol did not change over time (*F* (2,584) = 2.43, *p* = .089, η^2^ = .008). In addition, interaction between time and information type was not significant (*F* (2,584) = .34, *p* = .715, η^2^ = .001). Again there were no differences between school levels (*p*’s > .05). Because there was no change in intention to drink alcohol in both conditions, hypothesis 3 was rejected.

#### Additional analyses

Given the lack of differences between conditions we examined differences between conditions for students’ awareness of persuasive intent and counterarguing. Results showed that most participants were aware of the persuasive purpose of these materials, no matter whether narrative (*M* = 3.13; *SD* = .98) or non-narrative information (*M* = 3.03; *SD* = 1.00) was presented (*p*’s > .05). Students also engaged in little counterarguing, regardless of being in the narrative (*M* = 1.86; *SD* = .89; range: 1- 4) or non-narrative condition (*M* = 1.86; *SD* = .91). The mean scores on counterarguing did not differ significantly (*p*’s > .05) between conditions of school levels.

## Discussion

The aim of this study was to examine whether the learning and persuasive effects of an existing preventive school alcohol intervention could be increased by replacing the non-narrative information from existing materials with narrative information. We expected that non-narrative information would have a stronger learning effect than narrative information. For attitude towards alcohol and intention to drink alcohol, on the other hand, we expected that narrative information would have a stronger effect than non-narrative information. The results showed that there were immediate effects on knowledge and attitude towards alcohol, both in a more healthy direction, but these did not differ between conditions and did not persist over time. For intention to drink alcohol we did not find any significant effects.

Our current results suggest that both types of information have an equally strong immediate learning effect. These findings contradict the outcomes of previous studies conducted amongst adolescents [16, 17], on which our first hypothesis was based. These studies found that non-narrative information had a stronger learning effect than narrative information. Land and colleagues [16] suggested the interesting but irrelevant seductive details in narrative information to cause this difference between conditions in her study. Therefore, a potential explanation for the lack of a difference between conditions might be that there were no sufficiently interesting but seductive details in our narrative that could take attention away from the relevant knowledge. Consequently, we wondered what factors determine whether information about the context and characters in narrative information will be seductive details. One determinant may be the level of prominence in the narrative of the information that is targeted in the knowledge items of the questionnaire. This information may have been more prominent in our study than in Land’s study. In our study, the knowledge questions targeted the outcomes of the behaviour of the characters in the narrative. Because these outcomes were at the core of the plot, the targeted knowledge was prominent in the narrative information. In the study by Land and colleagues [16], on the other hand, the knowledge questions often asked about smaller details, like comments that characters made during a conversation in which also several other comments were made. As a result, the targeted knowledge was less related to outcomes of the actions of characters and less central in the plot. Therefore, it may have been harder for the students to determine which details in the narrative were relevant.

Although this study focused particularly on main effects, it is important to notice that previous research had suggested that narrative and non-narrative texts are processed differently in terms of the application of prior knowledge [30] and effects can be moderated by readers prior knowledge [29]. Within the context of the intervention that we focused on, it was not possible to distinguish between students with high and low prior knowledge. Hence, the finding of previous research that narrative texts are more effective for low prior knowledge students and non-narrative texts for high prior knowledge students was not applicable in this study [29]. It may however be of interest in contexts where it is possible to target high and low prior knowledge readers with different texts.

In addition to the learning effects, we examined whether the persuasive advantage shown for narratives in an education-entertainment context could be transferred to a school education context. As the results showed, there was no difference in effects on attitude and behavioural intention between conditions. Because of the educational context students may not have processed the narrative information with the purpose to get entertained, but in the same manner as they processed non-narrative information. Slater and Rouner [32] have suggested that this may occur when people are aware of the persuasive intent of a narrative, which was the case amongst our participants. Hence, we conclude that in a school context narrative information in health education materials is not likely to have a stronger persuasive effect than non-narrative information, because in this context the persuasive intent is probably more obvious to most students.

Furthermore, narrative information is suggested to be particularly beneficial for persuasive effects when the target group is likely to hold beliefs that contradict the message. Such groups are particularly likely to be resistant to the message. If people process narrative information with the goal to get entertained, they are supposed to generate less arguments against the message and, consequently, to be less resistant than if they would receive non-narrative information [32]. Hence, narrative information could be expected to be more effective than non-narrative information when the message is counterattitudinal. The students in our sample, however, engaged in little counterarguing, regardless of being in the narrative or non-narrative condition. The unfavourable alcohol attitude and low intention to drink alcohol scores at the baseline measurement suggests that students in our population hold beliefs, which are in line with the information in health education. Therefore, it was not likely that the students in our sample would be resistant, no matter the information type that was used. In a situation like this narrative information will not provide a persuasive advantage, which may also have accounted for the lack of differences between the conditions in this study.

A limitation of our study was the difference in text length between conditions. Narrative information typically is lengthier than non-narrative information because it contains background information about, for example, the context and the characters. This additional information is necessary to create a narrative, but makes it impossible to compare it to a non-narrative format without having a length difference. Of course, the non-narrative format could be lengthened as well, but this would either add additional new information or introduce a dose effect due to repetition of the same arguments.

The findings of this study may be limited in their generalizability, because this study was conducted in the context of low educated adolescents. It could be that findings will differ for populations with higher intelligence. In addition, it was not possible for us to draw a random sample of schools to include. Instead we were only able to include the schools that agreed to participate. As we have mentioned before, schools’ willingness to participate is typically low, because they receive many requests for participation in research and have limited time to do so. However, we have no reason to suspect that the schools that participated are different from schools that declined the invitation to participate. Hence, we do not believe this has harmed the generalizability of our findings, but we have to tread this issue with caution.

### Implications

Based on the explanations we offer for our findings we have three suggestions for future studies. First, research should focus on the characteristics of narratives that influence whether relevant information will be recognized as such or not. As we have argued above, the level of prominence of information that is targeted in knowledge questions may be one of these determinants, but this should be further tested. As a result, we may gain more insights into how people learn from narrative information and more understanding about the use of narrative information in health education materials. Second, future studies should examine the goals students have while they process narrative information in educational materials and whether this influences the effects. For example, if students read narrative information with the goal to get entertained, they may generate less counterarguments than they would if their goal was to get informed. Therefore, students’ processing goals is an important factor to consider. Third, there is currently little understanding about the effect of narrative and non-narrative information formats in situations where the message is proattitudinal. As we have mentioned before, previous studies suggested narrative information to be particularly beneficial in situations where the message is counterattitudinal. However, as proattitudinal non-narrative messages are found to be useful for reinforcing existing attitudes [42], this may as well be true for proattitudinal narrative information. Through the reinforcement the strength of the existing attitude may increase. Attitudes that are more strongly held, are found to be more persistent over time and have a stronger impact on behaviour [43]. However, such reinforcement effects may be examined more effectively through measures of attitude strength instead of attitude valence [44]. Because we did not include measures of attitude strength in our questionnaire, we cannot provide any insights into whether narrative or non-narrative information is more effective for reinforcing existing attitudes. Hence, researchers should conduct comparative research in which they examine the impact of proattitudinal narrative and non-narrative information on attitude strength. Such studies could reveal whether narrative information can also be applied for reinforcing existing beliefs and how this effect relates to the impact of proattitudinal non-narrative information.

This study also has some implications that professionals should consider when they develop health education materials about alcohol for low educated students. Based on our current findings it is not possible to advise either the narrative or the non-narrative information format to establish stronger learning effects. For persuasive effects, we have suggested that the processing goal to get informed, instead of being entertained, that students have in school inhibited the persuasive advantage of narrative information. We have also discussed that this processing goal is likely to be influenced by students’ awareness of the persuasive intent. Therefore, when selecting an information format for health education materials, developers should consider students’ awareness of the persuasive intent and whether they could be expected to process narrative information with the goal to get entertained. If students are not likely to have the goal to get entertained, then the selection of an information format could be based on other criteria then effectiveness. As we have mentioned in the introduction, low educated students are expected to perceive narrative information to be more enjoyable and less of a burden to process. Therefore, possible criteria could be the appreciation of an information format and the required effort to process information in a particular format. Insights into these criteria could be obtained by pretesting different formats during the development process.

From a policy perspective, it is also important to consider the costs for the developing materials. Narrative materials are typically more expensive to develop than non-narrative materials and here should be an increase in effectiveness that merits such additional costs [9]. From this point of view, the current results may be perceived as an argument not to favour the financing of materials containing narrative information instead of materials containing non-narrative information. Other criteria may nevertheless make policy makers favour materials containing narrative information. For example, if narrative information is more appreciated, this could result in higher self-administered exposure, which is also important to consider.

## Conclusion

We believe our study makes an important contribution to the existing knowledge about health education materials. No previous studies on health education materials have examined the effects of different types of information in the context of school health education about alcohol. We provide important insights by showing that both narrative and non-narrative information can be expected to have a similar immediate effect on knowledge and attitude for the topic of alcohol. These results, and the findings of previous studies (e.g, [10, 45]), raise the question whether narrative information can always be expected to be superior over other information formats as is suggested by scholars from the field of narrative persuasion [9]. We have offered theoretical suggestions to establish a more nuanced perception of the conditions under which narrative information can be expected to be more effective than non-narrative information. Examining our suggestions in future studies will help to further extend the existing knowledge about different information types in health education and the possibilities to develop effective materials for low educated adolescents. In addition, we have considered the implications of our findings for policy decisions, which will help to make more informed choices about financing more expensive materials containing narratives.

## Abbreviations

ES: Effect size
IQ: Intelligence Quotient
